# Can Extracellular Vesicles as Drug Delivery Systems Be a Game Changer in Cardiac Disease?

**DOI:** 10.1007/s11095-022-03463-z

**Published:** 2022-12-28

**Authors:** Akihiko Okamura, Yusuke Yoshioka, Yoshihiko Saito, Takahiro Ochiya

**Affiliations:** 1 Department of Molecular and Cellular Medicine, Institute of Medical Science, Tokyo Medical University, 6-7-1 Nishishinjuku, Shinjuku-Ku, Tokyo, 160-0023 Japan; 2 Department of Cardiovascular Medicine, Nara Medical University, 840 Shijo-Cho, Kashihara, Nara 634-8522 Japan

**Keywords:** cardiac disease, drug delivery system, exosome, extracellular vesicles, nucleic acids

## Abstract

Cardiac diseases such as myocardial infarction and heart failure have been the leading cause of death worldwide for more than 20 years, and new treatments continue to be investigated. Heart transplantation, a curative treatment for severe cardiac dysfunction, is available to only a small number of patients due to the rarity of donors and high costs. Cardiac regenerative medicine using embryonic stem cells and induced pluripotent stem cells is expected to be a new alternative to heart transplantation, but it has problems such as induction of immune response, tumor formation, and low survival rate of transplanted cells. On the other hand, there has been a focus on cell-free therapy using extracellular vesicles (EVs) due to their high biocompatibility and target specificity. Exosomes, one type of EV, play a role in the molecular transport system *in vivo* and can be considered a drug delivery system (DDS) innate to all living things. Exosomes contain nucleic acids and proteins, which are transported from secretory cells to recipient cells. Molecules in exosomes are encapsulated in a lipid bilayer, which allows them to exist stably in body fluids without being affected by nuclease degradation enzymes. Therefore, the therapeutic use of exosomes as DDSs has been widely explored and is being used in clinical trials and other clinical settings. This review summarizes the current topics of EVs as DDSs in cardiac disease.

## Introduction

Despite more rigorous management of risk factors such as smoking, dyslipidemia, and hypertension, cardiovascular diseases such as myocardial infarction (MI) and heart failure (HF) have been the leading cause of death worldwide for more than 20 years [1]. Endovascular catheterization and bypass surgery for ischemic heart disease or drug therapy such as β-blocker and ACE-I for heart failure with reduced ejection fraction (HFrEF) have been reported to improve prognoses; however, they are not curative treatments. Once cardiomyocytes become necrotic, they cannot regenerate and are eventually replaced by fiber tissue. As a result, it can develop into events that worsen the prognosis, such as cardiac dysfunction and fatal arrhythmias. Numerous preclinical studies have reported that cardiac regenerative therapy by embryonic stem cells (ESCs) and induced pluripotent stem cells (iPSCs) can repair and replace damaged blood vessels and cardiac tissue, followed by improved cardiac function. These stem cell-based therapies are expected to be a new curative treatment alternative to heart transplantation [2–4]. However, cell-based therapy has some problems, such as the induction of innate and adaptive immune responses, the possibility of tumor formation, and the low survival rate of transplanted cells [4, 5]. Therefore, cell-free therapy by extracellular vesicles (EVs) has attracted attention due to their high biocompatibility and target specificity [6, 7].

EVs are vesicles composed of lipid bilayers budding out of almost all cells [8]. EVs have membrane proteins and glycolipids on their surface layer, and various proteins and nucleic acids, including mRNA and miRNA, are present inside them [9, 10]. When first discovered, EVs were thought to be "trash cans" for secretory cells. However, it was discovered that EVs facilitate communication with other cells by transporting their contents from one cell to another [11, 12], and it is now clear that EVs play important roles in various biological phenomena, such as immune responses and signal transduction [13, 14]. Recently, research on the utilization of EVs for the diagnosis and treatment of diseases has been rapidly progressing. EVs contain nucleic acids, including miRNA, which are involved in the regulation of transcription and the expression of proteins and genes, and their composition is dependent on the cells that secrete them [15]. Many bioactive substances inside EVs have been reported to have therapeutic effects on various diseases, and EVs themselves are attracting attention as new biopharmaceuticals [16]. Furthermore, in some points, EVs have more suitable properties for drug delivery than synthetic nanoparticles and are expected to be a new drug delivery system (DDS) nanocarrier [17]. For example, the patient's own EVs as carriers are less immunogenic and more biocompatible than synthetic nanoparticles because the composition of the membrane is derived from autologous cells [18]. In addition, EVs may also have the ability to deliver molecules to specific cells in remote organs and tissues [19]. EVs with smaller sizes are also thought to have advantages such as avoidance of phagocytosis by monocytes, ability to migrate outside of blood vessels, passive accumulation in tissues, and permeability to barriers that exist *in vivo*, such as the blood‒brain barrier [20, 21]. EVs are also attractive because they possess a fusion mechanism with the plasma membrane that allows them to efficiently transport substances to the cytoplasm. Currently, studies on the delivery of nucleic acid drugs such as siRNA, protein drugs, and small molecule drugs using these EVs as carriers are underway [22–24].

In this review, with hopes for EVs serving as DDSs in the field of cardiac disease treatment, we focus on the therapeutic effects of EVs in several cardiac diseases and recent results demonstrating EVs as DDSs.

## What Are EVs?

**Fig. 1:**
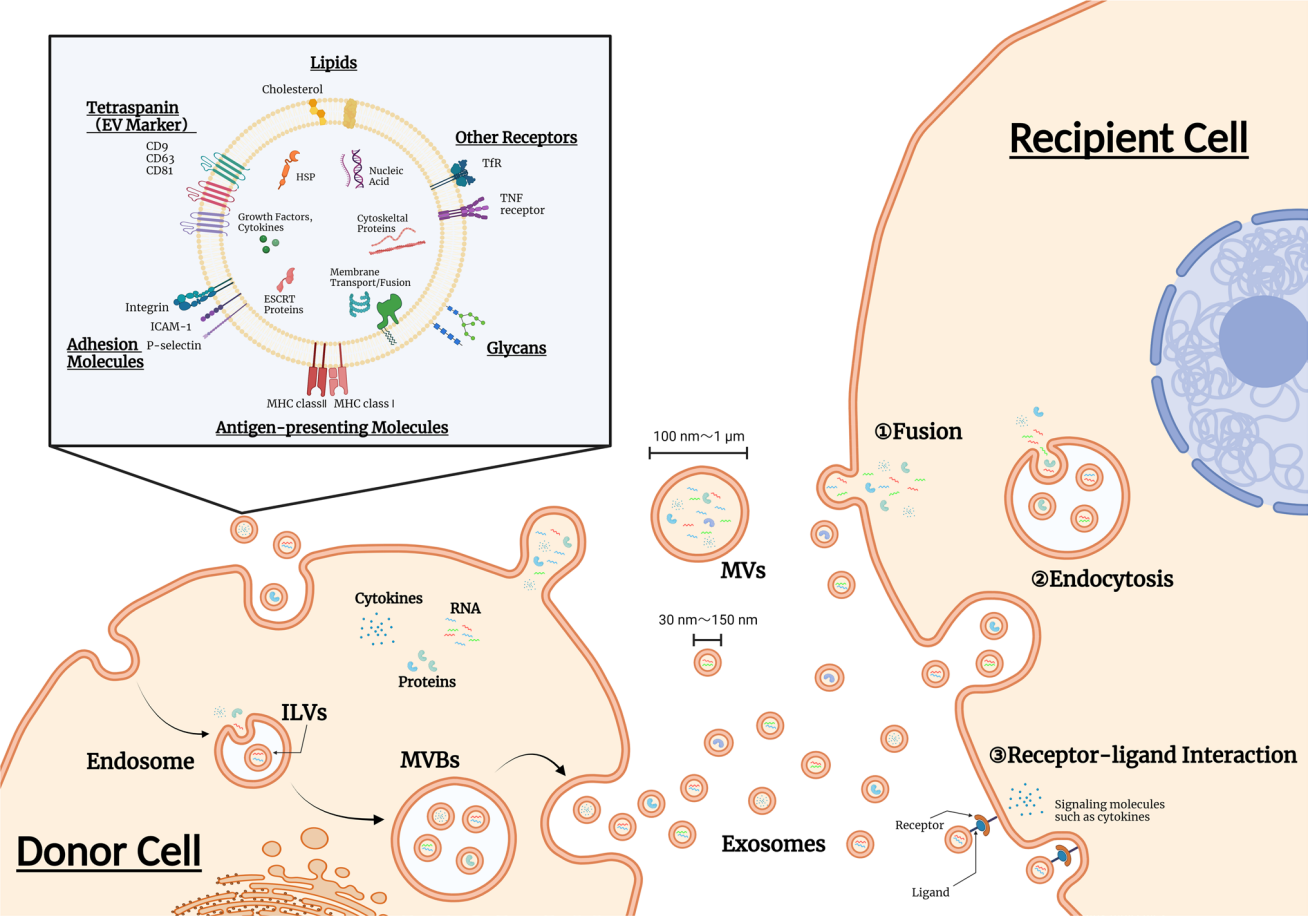
Biogenesis of EVs and mechanisms of intercellular communication by EVs. Exosomes, one of EVs, generally have a diameter of approximately 100 nm, and they are generally thought to be derived from endosomes. Endosomes are formed by endocytosis, entrap receptors on the plasma membrane, and are internally constricted to form intraluminal membrane vesicles (ILVs). Multivesicular bodies (MVBs) containing many of these ILVs fuse with the plasma membrane and are released into the extracellular space. Exosomes then play a role in mediating intercellular communication through endocytosis, fusion, or receptor-ligand interaction mechanisms. EVs have membrane proteins and glycolipids on their surface layer, and various proteins and nucleic acids, including mRNA and miRNA, are present inside them. The figure was prepared using BioRender (www.biorender.com). EVs: extracellular vesicles, MVs: Microvesicles, HSP: heat shock protein, ESCRT: endosomal sorting complex required for transport.

EVs are a generic term for particles naturally released from almost all cells that are delimited by a lipid bilayer [8]. According to the International Society for Extracellular Vesicles (ISEV), various subtypes are defined, such as endosome-origin “exosomes” and plasma membrane-derived “ectosomes” (microparticles/microvesicles) [8, 25]. No consensus has yet been reached on specific markers for EV subtypes, but some classifications are recommended. For example, EVs may be classified by size, with "small EVs" (sEVs) being defined as < 100 nm or < 200 nm and "medium/large EVs" (m/lEVs) being defined as > 200 nm [8]. Among them, exosomes are the main intercellular communication tools Fig. 1. Exosomes generally have a diameter of approximately 100 nm [26], and they are generally thought to be derived from endosomes [19]. Endosomes are formed by endocytosis, entrap receptors on the plasma membrane, and are internally constricted to form intraluminal membrane vesicles (ILVs) [27, 28]. Multivesicular bodies (MVBs) containing many of these ILVs fuse with the plasma membrane and are released into the extracellular space, where they are thought to serve as exosomes [29, 30]. Nanosized vesicles have attracted much attention in medical applications of EVs, such as applications in DDSs and therapeutic drugs, with exosomes playing a key role in particular. EVs contain various proteins, DNA, miRNA, and other nucleic acids [9, 10], which circulate in our body fluids and deliver their contents to target tissues and organs. There are two uptake mechanisms for EVs: ligand‒receptor-mediated uptake via molecules on the EV membrane and endocytosis. In the ligand‒receptor-mediated uptake mechanism, the tetraspanin family (CD9, CD81, Tspan8, etc.) and the integrin family, which are recognized as marker proteins on EV membranes, play a major role [31–34]. Surface proteins are thought to acquire their targeted delivery and organ specificity through their specificity for cell surface receptors of specific organs and tissues [35, 36]. As evidence of uptake by endocytosis, it has been reported that reduced uptake of EVs occurs at low temperatures and under the administration of endocytosis inhibitors *in vitro* [37]. Specifically, exosome uptake has been confirmed in various endocytic pathways, including clathrin-dependent endocytosis, macropinocytosis, and phagocytosis [38–42]. Exosomes play important roles in various biological phenomena occurring between cells, tissues, and individuals. Understanding the mechanisms of their production and secretion, as well as the characteristics of their contents in vesicles, can lead to the development of technologies to encapsulate nucleic acids and drugs in exosomes as carriers for DDSs.

## The Process for EVs to Function as DDSs

### Isolation and Purification of EVs

EVs have been found in body fluids such as blood [43], urine [44], saliva [45], ascitic fluid [46], pleural fluid [47], cerebrospinal fluids [48], and amniotic fluids [49]. Some isolation and purification methods for the use of EVs are described below.

#### Ultracentrifugation

The most common method to remove extraneous substances in cell culture supernatants or blood samples is stepwise centrifugation [8]. There are three types of ultracentrifugation (UC): the pellet down method, sucrose cushion method, and density gradient centrifugation, and the method is selected according to the purpose of the analysis. The pellet-down method is the simplest type of UC, in which particles of a certain size and density present in the solution are settled by centrifugation. Various centrifugation processes have been suggested, e.g., > 100,000 × g for 70 min has been used [50]. While UC is simple to perform and suitable for the isolation of relatively large sample volumes, whatever its vesicular or non-vesicular nature, i.e. whole or near-whole concentrated secretome. The methods of EV recovery are classified as high or low in the specificity section, and the simple ultracentrifugal recovery method is classified as low specificity [8].

#### Polyethylene Glycol Precipitation

This method uses polyethylene glycol (PEG) as a precipitant. EVs can be isolated by mixing the sample with PEG, allowing it to stand, and then centrifuging. The advantage of this method is that it does not require an ultracentrifuge or expensive special devices. The method is as simple as UC. However, the size range of the isolated products is wide, and there is a possibility of contamination by proteins or EVs larger than exosomes [51].

#### Immunoprecipitation

The immunoprecipitation (IP) isolation method uses antibodies that recognize EV membrane proteins. Since the IP method is based on an antigen–antibody reaction, it is possible to isolate EVs with high purity and specificity [51]. However, it is difficult to isolate EVs for which a target antigen has not been found, making it difficult to use for unknown samples.

In addition to the above, there are several other purification methods for EVs. For example, there are size-exclusion chromatography (SEC) methods, tangential flow filtration, separation by biophysical properties such as surface charge or acoustic, and membrane-affinity columns. However, current investigations of EVs are focused on basic aspects such as the more convenient method for EV isolation (mainly SEC and UC) [52]. Currently, most of the studies on EVs are reported in laboratory cell and animal experiments, therefore, the purity and recovery efficiency of SEC and UC are well-tolerated and convenient for such studies. However, for clinical application, it is very important to isolate a large amount of EVs that are safe when administered *in vivo*. At present, SEC and UC are not sufficient for clinical applications regarding EV recovery and purity. We think that, to advance the clinical application of EVs, new technological innovations in recovery methods will be essential. In fact, in the preclinical stage at the laboratory level, cultured cell-derived EVs are often used in research because they are relatively easy to handle and a stable supply of EVs of constant quality can be easily obtained, but the problem is that only a small number of EVs can be isolated. Currently, several strategies are being investigated to solve this quantitative problem of EV isolation [53]. For example, Watson *et al*. used a hollow fiber culture system, which enabled continuous production of EVs and increased EV production by more than 40-fold compared to conventional cell culture techniques [54]. Others have reported a more than 100-fold increase in EV production by applying mechanical extrusion [55]. Although these reports have been successful in recovering large numbers of EVs, physical stress is applied to the EVs during recovery, and attention must be paid to the deformation of the EVs themselves and changes in membrane electric potential. Another point of view, Umezu *et al*. reported that exosome-like vesicles obtained from the plant acerola juice can be stably supplied at a low cost. Acerola-derived vesicles can be orally administered to mice, and the accumulation of EV inclusions in target organs has been confirmed [56]. Isolation and purification of EVs are thought to be the most important issues to be solved to advance the clinical application of EVs. In addition to the above, many technological innovations have suggested new purification methods for EVs, but the safety of the EVs collected should always be carefully confirmed for clinical application.

### Loading Pharmaceuticals into EVs

EVs affect receptor cells by delivering encapsulated nucleic acids and proteins, etc., and several methods have been developed to encapsulate pharmacologically active substances within EVs. These methods can be divided into two main categories. One is the preloading method, in which the EV-producing cells are modified before EVs are isolated, and the other is the postloading method, in which the isolated EVs are loaded with the drug by chemical or physical means.

#### Preloading Method

The preloading method is a method in which cells that are the source of EVs are genetically modified to strongly express a target protein or miRNA and encapsulate it in secreted EVs; alternatively, cells are cultured in a medium containing a compound to be delivered, the cells are allowed to take up the compound, and the EVs encapsulated in the compound are secreted [57–60]. The molecular sorting mechanism for EVs has been partially understood, and it has been reported that the RNA-binding proteins hnRNPA2 B1 [61], Y-box protein 1 [62], and annexin 1 [63] are involved in the sorting of miRNA into EVs. It has been reported that EVs containing high levels of target proteins can be isolated by modifying the target proteins to promote their uptake by EVs and then expressing them at high levels in donor cells [63–65].

#### Postloading Method

A postloading method involves directly processing EVs and loading them with therapeutic molecules such as miRNA. This method is relatively easy to use compared to preloading and is widely used today.

In postloading, the most commonly used method at present is electroporation, among which there are many reports of siRNA internalization [66]. Wahlgren *et al*. successfully introduced siRNA into EVs and delivered nucleic acids to monocytes and lymphocytes, but it requires optimization of the voltage, capacitance, range between electrodes in the cuvette, and concentration of siRNA and EVs [67]. While this method can be applied to any EV, there are concerns that RNA and EVs may aggregate due to the applied electric field [68].

Coincubation is used to efficiently load therapeutic molecules such as siRNA into EVs without altering vesicle size distribution or integrity [69, 70]. Didiot *et al*. reported that exosomes loaded with siRNA targeting Huntingtin mRNA were efficiently internalized by mouse primary cortical neurons and promoted dose-dependent silencing of Huntingtin mRNA and protein [70].

Sonication is a method of allowing drugs to permeate membranes by temporarily disrupting membrane integrity through sonication [71, 72]. Lamichhane *et al*. reported that EVs loaded with therapeutic siRNA via sonication were taken up by recipient cells and were capable of target mRNA knockdown, leading to reduced protein expression [71]. However, Haney *et al*. confirmed that EVs obtained by sonication were nonspherical EVs with structural changes [71]. It has also been reported that membrane integrity is isolated by incubation at 37°C for 1 hour [73].

Various other postloading methods have been proposed, including mechanical extrusion [71, 74], freeze/thaw cycles [75–77], and RNA loading using a commercial Exo-Fect (chemical transfection) kit [78–81]. For example, there are reports of loading more inclusions by using saponin, a surfactant molecule, which forms a complex with cholesterol in the lipid membrane to create pores and improve the permeability of hydrophilic molecules. Fuhrmann *et al*. reported that the saponin-assisted method, in particular, allowed up to 11-fold higher drug loading of hydrophilic porphyrins compared to passive methods [74]. However, saponins have also been reported to have *in vivo* hemolytic activity [82]. Thus, drug loading into EVs has been attempted by various methods, but careful consideration is needed to ensure that the isolated EVs with encapsulated drugs are safe for therapeutic applications.

### Target Specificity of EVs

EVs are expected to have a target specificity originally to their donor cells because they have many of the similar surface proteins of their donor cells. Thus EV surface proteins are specific ligands for recipient cell surface receptors, e.g., integrin family, etc. [34]. In fact, however, it has been shown that when EVs are administered to living organisms, most of them accumulate in the liver and spleen, as with ordinary liposomes, and are eventually cleared by macrophages in these organs [83, 84]. Therefore, there is a need to develop methods for the efficient delivery of EVs to target tissues by further enhancing their targeting ability. Alvarez-Erviti *et al*. successfully expressed a protein called the RVG peptide on the membrane. This RVG peptide is a neuron-selective protein that binds to acetylcholine receptors, and its expression on the EV membrane enables specific delivery to neurons [85]. Nakase *et al*. used crosslinkers to modify oligoarginine peptides against EV membrane proteins. The oligoarginine peptide modification allowed the active induction of macropinocytosis without cytotoxicity, thereby dramatically increasing the efficiency of EV uptake into target cells [86]. In this report, the efficacy of the cytosolic release of contents from EVs inside cells was not assessed. To achieve effective pharmacological effects in target cells, in addition to EV uptake, it is also necessary to focus on techniques that enhanced the cytosolic release of EV contents.

Magnetic, inorganic materials can be used for noninvasive treatment since their strength and direction can be easily controlled, enabling targeting to specific sites and improving delivery efficiency [87]. In particular, superparamagnetic iron oxide nanoparticles (SPIONs) have excellent magnetic properties and are used as magnetic nanoplatforms for targeted drug carriers [88]. However, there are still many unknowns about toxicological considerations concerning the use of SPIONs as drug delivery vehicles and such a magnetic field can only be used in the hospital setting. In addition to those strategies of modifying the EVs themselves to improve target specificity, there are also reports of combining hydrogels and EVs to improve local retention [89]. For example, Lv *et al*. confirmed the prolonged therapeutic efficacy of EVs incorporated into alginate hydrogels in a mouse model of myocardial infarction [90]. Other reports have shown that combining EVs with various biomaterials enhanced target specificity and improved therapeutic efficacy, the details of which are described in later chapters [91–93].

### Therapeutic Effects of Non-modified EVs on Cardiac Disease

Generally, the biological functions of EVs depend on the state of the donor cells and can change during different microenvironments [15]. For example, EVs derived from bone marrow-mesenchymal stem cells (BM-MSCs) induce cardioprotective effects similar to those of their parental BM-MSCs and reduce inflammation induced by macrophages after myocardial infarction [94]. This chapter will focus on the efficacy of EVs, especially their endogenous miRNA, on target diseases and even physiological phenomena.

### Myocardial Infarction

MI is a disease in which a coronary artery that nourishes the myocardium is blocked, resulting in necrosis of the myocardium due to lack of oxygen. The key to the treatment of MI is to rescue and protect myocardial cells, which once necrotic, will never regenerate. Once cardiomyocytes develop ischemia, inflammatory macrophages immediately accumulate and begin phagocytosis of the necrotic cells. Eventually, the spaces that once had cells are filled by fibrosis, which leads to lethal arrhythmias and reduced cardiac function. In addition, once occluded coronary arteries may recanalize, a large number of reactive oxygen species (ROS) flow into distal tissues, leading to necrosis of cardiomyocytes that could still be viable. This phenomenon, called ischemia/reperfusion injury (I/R injury), is also important in understanding myocardial damage caused by MI. Furthermore, as is generally observed in all tissues, the myocardium begins angiogenesis from the time it is exposed to ischemia to resist the lack of oxygen. EVs are involved in each of these processes: inflammation, fibrosis, I/R injury, and angiogenesis. In recent years, the efficacy of MSC-EVs has been widely reported, but there have been other reports of cardioprotective effects of EVs derived from various cell types.

#### Inflammation

Myocardial ischemia or inflammation causes myocardial cell loss, which in turn leads to replacement fibrosis in an attempt to maintain the myocardial tissue architecture. Following fibrosis, the electrophysiological integrity of myocardial tissue is also impaired, which is known to trigger arrhythmias and cause diastolic dysfunction due to increased stiffness. Using animal models of MI, Biemmi *et al*. reported that circulating EVs carrying IL-1α and IL-1β were increased and that the reduction of these inflammatory EVs improved cardiac function [95]. Inflammation is considered the most important phenomenon in the progression of MI, and therefore, there have been a tremendous number of reports focusing on it. In addition, there are many types of cells that secrete EVs, and differences in the origins of such secretory cells are important in EV research. The following are representative reports of EVs that have been shown to have anti-inflammatory effects on several recipient cells.

Zhao *et al*. reported that BM-MSC-derived EVs (BM-MSC-EVs) maintain anti-inflammatory macrophage phenotype polarity in a mouse MI model via miRNA-182 delivery, which has important effects on cardiac repair, inhibition of toll-like receptor 4 (TLR4) in recipient cells, and immune regulatory properties [96]. EVs derived from adipose tissue-derived mesenchymal stem cells (AD-MSCs) also have cardioprotective effects through anti-inflammatory effects. In a rat MI model, AD-MSC-EVs induced anti-inflammatory macrophage polarization and reduced serum levels of IL-6, IL-1β, TNF-α, and IFN-γ. As a result, cardiac function improved, and collagen fiber accumulation was reduced [97]. AD-MSC-EVs were also effective in inhibiting myocardial infarct expansion and apoptosis and simultaneously reduced serum levels of myocardial injury-specific markers [98]. AD-MSC-EVs exerted cardioprotective effects in cardiomyocytes through the Wnt/β-catenin signaling pathway after myocardial I/R. However, this study did not clarify how AD-MSC-EVs affect Wnt/β-catenin signaling and whether some molecules secreted by AD-MSC-EVs are involved in this process, such as miRNAs and proteins. Human umbilical cord mesenchymal stem cell-extracellular vesicles (hUC-MSC-EVs) also affected fibroblast (FB) phenotypic differentiation and function during the inflammatory phase after MI, promoting FB-to-myofibroblast (MFB) transition. They suppress inflammation and protect cardiomyocytes. This mechanism was reported to be anti-inflammatory by decreasing the expression of miRNA-125b-5p, which is normally upregulated in patients with acute MI, and by promoting Smad7 expression. [99, 100]. In this study, they hypothesized that hUC-MSC-EVs might contain a competing endogenous RNA, for example, lncRNA, which might competitively bind miRNA-125b-5p to facilitate Smad7 expression in cardiomyocytes. However, how hUC-MSC-EVs induce miRNA-125b-5p-mediated downregulation remains unknown.

Reports of EVs derived from MSCs are still the most common, with the second most common being reports of cardiosphere-derived cells (CDCs) serving as secretory cells. CDC-EVs have also been reported to have anti-inflammatory effects. In a rat MI model, treatment with CDC-EVs led to decreased expression of inflammatory genes such as Nos2 and Tnf. After 48 h of MI, CD68 + macrophages were reduced in the infarct border tissue. miRNA-181b in CDC-EVs is transferred to macrophages, reducing PKCδ transcript levels. This has been reported to alter the phenotype of macrophages and exert a cardioprotective effect [101]. It has also been reported that miRNA-126 is also contained in CDC-EVs, which, in addition to protecting from inflammation, protects cardiomyocytes from apoptosis, fibrosis, and angiogenic damage [102].

iPS cells have shown their efficacy in stem cell therapy, and cardioprotective effects have also been reported in EVs from iPS cells. Recently, Correa *et al*. reported that in a mouse model of chronic and acute MI, treatment with iPS-cardiac progenitor cell-extracellular vesicles (iPS-CPC-EVs) induced immune-related signaling pathways and promoted tissue repair in damaged hearts. In an acute model characterized by a strong inflammatory response, the number of neutrophils decreased, the expression of inflammatory cytokines such as IL-1α, IL-2, and IL-6 decreased, and anti-inflammatory IL-10 increased. In a chronic model, inflammatory monocytes and cytokines, namely, IL-1α, IL-1β, TNFα, and IFNγ, were decreased in the MI area [103].

Many reports on the anti-inflammatory effects of EVs after MI have focused on the regulation of inflammatory macrophage polarity, which is also an important target in other inflammatory diseases. EVs derived from ischemic tissue and cells induced anti-inflammatory macrophage polarity and decreased the expression of inflammatory cytokines such as IL-1α, IL-2, and IL-6, suggesting that they may have anti-inflammatory properties.

#### Angiogenesis

To rapidly ameliorate the ischemic/hypoxic state of the infarcted myocardium, the endocardial endothelium is induced, and angiogenesis is initiated by the action of vascular endothelial growth factor (VEGF) and other factors secreted by cardiomyocytes. This section describes EVs and angiogenesis with a focus on secretory cells, mainly MSCs, CPCs, and stem cells (iPS and ES cells).

Wang *et al*. observed that BM-MSC-EVs injected intravenously into mice with MI recovered cardiac function by increasing vascular density through downregulation of Efna3, a target gene of miRNA-210. Zhu *et al*. reported that this BM-MSC-EV-derived miRNA-210 was involved in the reduction of infarct size and apoptosis of cardiomyocytes, activation of resident progenitor cells, and ultimately in the improvement of cardiac function, in addition to its angiogenic effect [104, 105].

Cardiomyocyte progenitor cells (CPCs) are considered pluripotent stem cells that differentiate into cardiomyocyte-like cells and vascular cells *in vitro* and have been used as a cardiac regeneration therapy by being transplanted into the infarcted heart [106–108]. CPC-EVs had cardioprotective effects similar to those of secretory cells and CPCs. CPC-EVs deliver endoglin, which activates endothelial cells and promotes angiogenesis [109], and the inner components of CPC-EVs were found to be enriched in proangiogenic miRNA, such as miRNA-146a-3p, miRNA-132, and miRNA-210 [110, 111].

Stem cell-derived EVs, such as those derived from iPS cells and ES cells, have been reported to have angiogenic effects similar to those of their parent cells, and several reports have suggested that they are superior to treatment with secretory cells themselves in several respects. Extensive transcriptomic and proteomic studies were performed on mouse FB-derived iPSC-EVs by Adamiak *et al*. The results showed that iPSC-EVs, as well as iPSCs, were enriched in miRNA associated with angiogenesis, adaptation to hypoxic stress, cell cycle regulation, and senescence processes. In particular, specific miRNA involved in cell proliferation, cell differentiation, apoptosis, and maintenance of self-renewal capacity and pluripotency, such as let-7, miRNA-145, the miRNA-17–92 cluster, and miRNA-302a-5p, were detected only in iPSC-EVs, not in iPSCs [112]. On the other hand, iPSC-EVs used in this study contained both sEVs and mEVs based on the size criteria [8]. Thus, there is a possibility that we are looking at various subpopulations of EVs with different formation pathways, such as exosomes and ectosomes, and the differences in pharmacological effects depending on the subpopulation of EVs should be investigated in more detail in the future.

Pluripotent stem cells widely used for cardiomyocyte therapy also include ESCs [113]. Injection of human ESC-cardiovascular progenitor cells (hESC-CVPs) into the subacute phase of I/R injury in a rat MI model improved cardiac function [114]. In addition, in the case of injecting hESC-CVP-derived EVs (hESC-CVP-EVs), similar results of improved cardiac function, reduced fibrotic scarring, and preserved myocyte size were observed [115]. Treatment with hESC-CVP-EVs showed a tendency to be superior to hESC-CVP treatment, especially in angiogenic capacity [115]. In addition, injection of EVs derived from hESC-CVPs exposed to normoxia and hypoxia into a mouse MI model showed improved cardiac function and reduced scar size at 28 days, with a greater effect on EVs derived from cells cultured under hypoxic conditions. EVs exposed to hypoxia have high levels of the lncRNA MALAT1, which increases miR-497 at target sites and promotes angiogenesis to improve cell viability [116]. It is necessary to proceed with research about ESCs while giving due consideration to the fact that the production and use of ESCs are basically limited to basic research, and the replication of human individuals by cloning is prohibited. Currently, we think that there are no clear regulations on ES cell-derived EVs, and therefore, there may rather have room for future development as a cell-free therapy.

Similar to inflammation after MI, ischemic tissues and cells promote angiogenesis to recover from hypoxia, and it was suggested that EVs were also involved in this process. EV therapy for angiogenesis may also be effective for ischemic heart disease such as angina pectoris, which is a preliminary stage of MI. It is expected that the effectiveness of EV therapy will be reported in the prevention of MI and the treatment of many ischemic diseases, not limited to MI.

#### Ischemia/Reperfusion Injury

I/R injury occurs after an organ experiences an ischemic injury, and then blood flow is resumed. I/R injury is considered to be caused by ROS produced at various sites that disturb mitochondrial function and by hypercontractile zone necrosis due to excessive intracellular Ca2 + influx.

Liu *et al*. reported that in a rat MI model, treatment with BM-MSC-EVs pre-reperfusion reduced reperfusion injury by inducing increased autophagy in cardiomyocytes via the AMPK and Akt pathways, reducing cell apoptosis and infarct size [117]. In their study, autophagy dysfunction is harmful, and moderate enhancement of autophagy is beneficial. In the future, further studies are needed to define the appropriate autophagy state. CDC-EVs are enriched with Y RNA, called EV-YF1, which has been reported to be protective against I/R injury; injection of EV-YF1 into the left ventricle of I/R injury rats reduced myocardial injury compared to controls. In addition, IL-10 expression was induced, and the effect was dependent on the concentration of EV-YF1 [118, 119].

I/R injury is a common occurrence, especially during the catheterization of MI. The use of these EVs together with catheterization may minimize reperfusion injury.

#### Fibrosis

Cardiac fibrosis results from the activation of cardiac FBs and deposition of excess extracellular matrix, leading to increased stiffness and reduced flexibility of the myocardium, causing myocardial contractile or diastolic dysfunction [120]. An important step in this pathological process of cardiac remodeling is the transition from cardiac FBs to MFBs, which is thought to occur during the progression of many cardiovascular diseases.

There have been numerous reports of miRNA associated with fibrosis after MI [121–123]. For example, miRNA-208a expression was upregulated in rat cardiomyocytes after MI, and this miRNA was delivered to FBs by EVs and involved in FB proliferation and myocardial fibrosis [121, 122]. It has also been reported that EVs derived from activated CD4 + T cells promote the activation of cardiac FBs by miRNA-142-3p-WNT signaling and promote cardiac fibrosis after myocardial ischemia [123]. Additionally, it has been reported that miRNA and other proteins in EVs modulate FB function and improve myocardial fibrosis [124–129]. miRNA-26a overexpression has been reported to regulate extracellular matrix production and inhibit myocardial fibrosis caused by MI [126–128]. Ibrahim *et al*. reported that CDC-EVs highly internalized miRNA-92a and attenuated myocardial fibrosis and improved survival in a mouse model of MI [129].

Fibrosis is the result of an inflammatory response and is a state of burnout. Fibrosis is an irreversible phenomenon; it is important to suppress or reduce fibrosis, and the EVs described above were thought to have this effect. Currently, catheterization and bypass surgery are practiced as effective treatments for MI. In addition, an early combination of EV therapy with antifibrotic effects is expected to have a greater cardiac function preservation effect by preventing fibrosis.

### Heart Failure

HF is a clinical syndrome caused by structural and functional abnormalities of the heart [130]. The clinical term "heart failure" can vary widely, including left HF or right HF [131] and ischemic or nonischemic HF. In the pathogenesis process, EVs have been shown to be involved in apoptosis, myocardial fibrosis, and angiogenesis [132].

EVs from HF patients showed a decreased amount of miRNA-21-5p. Treatment with HF patient-derived EVs (HF-EVs) in a mouse myocardial infarction model resulted in reduced cardiac function and worsened left ventricular remodeling. More detailed analysis showed that HF-EV-derived miRNA-21-5p promotes angiogenesis and cardiomyocyte survival via the PTEN/Akt pathway and cardiac repair via EVs [133]. In HF-EVs, mi-21-5p was decreased, suggesting that angiogenesis was also suppressed. However, the mouse model of heart failure which was used in this study was relatively young. Therefore, it must be considered that their endogenous reparative ability does not resemble that of aged humans whose innate repair systems may be compromised, especially those suffering long-term heart failure.

Hypertrophic myocardium is often the cause of poor cardiac function, and there have been reports on cardiac hypertrophy and EVs. In cultured cardiomyocytes, MSC-EVs also inhibited hypertrophy of cells stimulated with angiotensin II. In addition, treatment of mice with induced HF (TAC mice) with BM-MSC-EVs showed significant protection of the myocardium against cardiac hypertrophy, antifibrotic effects by promoting premature aging of cardiac FBs, and maintenance of cardiac function under pressure overload. These results suggest that BM-MSC-EVs have an inhibitory effect on cardiac hypertrophy and may be an effective therapeutic tool against cardiac remodeling by promoting apoptosis and antifibrotic effects [134].

In animal models of cardiac hypertrophy, EVs from hypertrophic cardiomyocytes were shown to activate macrophages by transferring miRNA-155 and induce adverse cardiac remodeling by releasing the inflammatory cytokines IL-6 and IL-8 [135, 136]. *In vitro*, FBs stimulated with TNF-α also release EVs with high expression of miRNA-27a, miRNA-28-3p, and miRNA-34a, inducing dysregulation of the Nrf2 pathway. The resulting oxidative stress disorders have been shown to promote cardiac remodeling and dysfunction [137]. Cardiac FBs secrete EVs containing miRNA-21 and miRNA-27a, while cardiomyocytes release EVs containing miRNA-217; all three are miRNAs that induce cardiac hypertrophy and are potential therapeutic targets for cardiac hypertrophy [138–140].

HF is the result of various diseases, and many aspects of its pathogenesis remain unknown. EVs can influence the microscopic communication between cells, which will lead to the elucidation of the pathophysiology of cardiac diseases and new therapeutic agents.

### Arrhythmia

The few studies involving EVs and arrhythmias mainly focus on pathophysiology and diagnosis. Several studies have been reported on the diagnosis of atrial fibrillation and one of its causes, atrial fibrosis [141–145].

As mentioned above, the most important step in fibrosis is the activation of cardiac FBs to MFBs. Paracrine factors released from MFBs modify ion channel expression in cardiomyocytes. Decreased expression of the L-type calcium channel Cav1.2 is a characteristic of atrial fibrillation-associated ion remodeling. Li *et al*. demonstrated that cardiomyocytes treated with MFB-derived EVs exhibit downregulation of the L-type calcium channel Cav1.2. They considered EV-mediated crosstalk between MFBs and cardiomyocytes, contributing to increased vulnerability to atrial fibrillation by reducing the expression of Cav1.2 in cardiomyocytes [141]. Yao *et al*. found that in beagles undergoing rapid atrial pacing for 7 days, the number of EVs in both the atria and plasma increased. Plasma EVs contained high levels of miRNA-21-5p, which was upregulated in the atria and was associated with decreased expression of its target, tissue inhibitor of metalloproteinase 3 (TIMP3), as well as increased expression of transforming growth factor-β1 (TGF-β1), collagen I/III, and matrix metalloproteinases. Furthermore, they reported that these effects in the atria were suppressed by inhibitors (GW4869) of EV generation, suggesting that miRNA in EVs is involved in part in the fibrosis progression process and that these EVs may be targets in the treatment of atrial fibrillation [142].

EVs contain not only miRNA but also a wide variety of proteins and cytokines, and there have been numerous reports of their therapeutic effects in the field of cardiovascular medicine. The reports of cardioprotective EVs listed here are just a few. However, current EV therapies are far from clinical application, and most of them are at the level of animal experiments. A major reason for this is the difficulty in reaching and maintaining a sufficient amount of EVs in the heart to achieve an effect. The greatest challenge to clinical application is how to increase the cardiac specificity of EVs. Hence, attempts to improve target specificity and retention in the heart are discussed in the next section.

## Reports of Modified EVs as DDSs in Cardiac Disease

As described above, EVs and EV-associated miRNA have been reported to demonstrate cardioprotective effects in cardiac diseases, and their high potential as a therapy for cardiac diseases has been recognized. However, it is also true that none of them are at the level of clinical application. The most important issues that need to be resolved are target specificity to the heart and local maintenance. To resolve this issue, creative efforts are being developed to further enhance the therapeutic effect on the heart, such as modifying EV inclusions, improving delivery to the myocardium, combining them with other tools, and investigating methods of administration, which are described below.

### Loading Specific Molecules into EVs

By the electroporation method described above, Ma *et al*. loaded BM-MSC-EVs with miRNA-132. miRNA-132 directly targets p120RasGap (RASA1) and regulates endothelial cell behavior during angiogenesis. Treatment of a mouse MI model with miRNA-132-loaded BM-MSC-EVs markedly enhanced peri-infarct angiogenesis and significantly increased the left ventricular ejection fraction [146].

Wang *et al*. similarly demonstrated the electroporation method to load MSC-EVs with miRNA-101a, a major inhibitor of fibrosis. Surprisingly, even though only 4% of the injected EVs reached the ischemic myocardium, they reduced infarct size and fibrosis and improved cardiac function while inducing anti-inflammatory effects [147].

Zhang *et al*. found that BM-MSC-EVs overexpressing miRNA-148a had a significant reduction in SPARC-associated modular calcium-binding protein 2 (SMOC2), thereby inhibiting the onset of atrial fibrillation and reducing cardiomyocyte apoptosis [145]. Thus, many reports have shown additional therapeutic effects by loading miRNA with additional specific cardioprotective effects into cardioprotective parental cell-derived EVs, such as those derived from BM-MSCs [145–147].

Cardioprotective additives within EVs are not limited to miRNA but also include a wide variety of natural compounds, transcription factors, cytokines, lncRNA, and many others. Curcumin, a natural compound, has both anti-inflammatory and antifibrotic effects [148–150]. Mixing curcumin with EVs allows curcumin to be self-assembled into the lipid bilayer of EVs, which may protect curcumin from degradation. In addition, the aqueous solubility and stability of curcumin are improved, resulting in increased bioavailability. Treatment of lipopolysaccharide-induced septic shock model mice with these curcumin-EVs effectively delivered curcumin to inflammatory cells and significantly reduced levels of inflammatory factor [151].

Xu *et al*. used an atrial fibrillation model and showed that treatment of rats with Nrf2 lentivirus-transduced BMSC-EVs significantly downregulated the Nrf2/HO-1 pathway and suppressed arrhythmias, myocardial fibrosis, apoptosis, and inflammation due to atrial fibrillation [144].

Zhang *et al*. obtained IFN-γ-loaded MSC-EVs by coculturing IFN-γ with MSCs. Then, IFN-γ-loaded MSC-EVs were used to treat a mouse MI model in the infarction region, increasing the amount of miRNA-21 and causing decreased fibrosis, reduced apoptosis of cardiomyocytes, and improved cardiac function [152].

In addition, X-inactive specific transcript (XIST), a type of lncRNA, was transfected into EVs derived from mouse adipose tissue-derived mesenchymal stem cells (AMSCs) and then injected into an atrial fibrillation mouse model. As a result, atrial fibrillation was suppressed. They suggested that XIST may blunt myocardial pyroptosis by absorbing miRNA-214-3p to promote Arl2 expression, providing encouraging insight into XIST-based targeted therapy for atrial fibrillation [143].

**Fig. 2:**
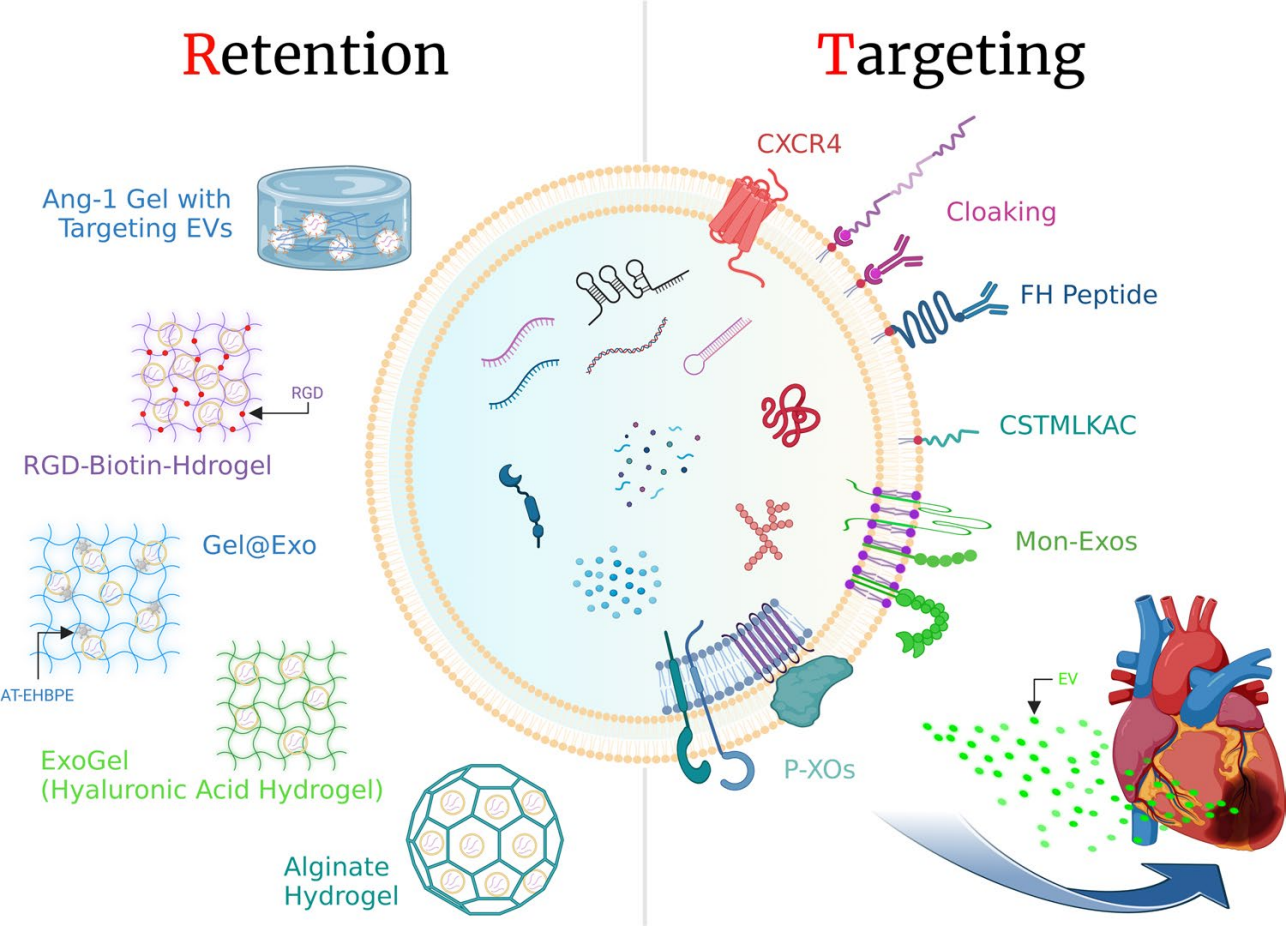
Retention and Target Specificity for the Heart. EVs mixed with improved gels are treated locally in the heart to improve retention in the heart (left). EVs are loaded on the membrane with proteins or antibodies specific to the myocardium to improve the targeting of EVs to the heart (right). The figure was prepared using BioRender (www.biorender.com). EVs: extracellular vesicles, RGD: arginyl-glycyl-aspartic acid, AT-EHBPE: aniline tetramer- hyperbranched epoxy macromer, Mon-Exos: monocyte mimic-bioinspired mesenchymal stem cell-EVs, P-XOs: platelet membrane hybrid exosomes.

In the following section, we discuss reports of EVs with improved cardiac targeting or cardiac retention to increase their therapeutic effect on the heart Table I and Fig. 2.

**Table I:** List of EV Therapies as DDSs for Cardiac Diseases in the Last 4 Years

Method	Cell source	Animal model	Improvements to increase the pharmaceutical effects	Therapeutic effects	Ref
homing peptide (cardiac, inflammation)	mesoporous silicon nanoparticles (MSNs)	MI/IR model mouse	coating FH peptide-modified neutrophil-mimicking membranes	improved cardiac function, attenuated fibrosis	[168]
homing peptide (cardiac, endothelial cell)	mesoporous silicon nanoparticles (MSNs)	MI model mouse	platelet membrane hybrid exosomes (P-Xos)	benefit the cardiac remodeling	[169]
homing peptide (cardiac)	nanoparticles	MI model rabbit/rat	nanoparticles bonds to two types of antibody binding either to CD63 antigens or to myosin-light-chain	reduced MI size, improved LVEF and angiogenesis	[167]
homing peptide (cardiac)	CPCs	IR model rat	CXCR4 overexpression on EV membranes	reducted MI size, improved LVEF	[161]
homing peptide (cardiac)	CDCs	neonatal mouse	cardiomyocyte specific peptide WLSEAGPVVTVRALRGTGSW on membranes	reductied scar size, preserved LVEF, reduced cardiomyocyte apoptosis	[170]
homing peptide (cardiac)	BM-MSCs	MI model mouse	targeting peptide CSTSMLKAC (IMTP) on EV membranes	attenuated inflammation and apoptosis, reduced fibrosis, enhanced vasculogenesis and cardiac function	[164]
homing peptide (cardiac, muscle)	CDCs	IR model rat	modular EV membrane anchoring platform (DMPE-PEG-STVDN; DPS) with cardiac homing peptide or antibody	reduced fibrosis and scar size, increased cellular proliferation and angiogenesis	[171]
homing peptide (cardiac)	CDCs	IR model rat	targeting peptide CSTSMLKAC (IMTP)	increased cardiac function, reduced cardiac fibrosis and apoptosis, increased cellular proliferation and angiogenesis	[165]
homing peptide (cardiac)	MSCs	MI model	cTnI-targeted exosomes	promoted cardiomyocyte proliferation in the peri-infarct area, restored cardiac function	[163]
homing peptide (endothelial cell)	BM-MSCs	MI/IR model mouse	monocyte mimic-bioinspired MSC-EVs (Mon-Exos)	improved cardiac function and pathohistology changes	[162]
hydrogel	MSCs	MI model	ISL1 overexpression MSC-EVs with Ang-1 gel	retentioned and enhanced the anti-apoptosis, proliferation and angiogenic capacity in endothelial cells	[156]
hydrogel	MSCs	TAC mouse/pig	MSC-EVs in hyaluronic acid (HA) hydrogel (ExoGel)	reduced LV chamber size, preserved wall thickness	[157]
hydrogel	iPS-CPCs/MSCs	MI model pig	EVs in MA–HA hydrogel	mitigated cardiac remodeling, improved cardiac function	[159]
hydrogel	MSCs	MI model rat	HIF-1α-MSC-EVs in RGD-biotin hydrogel	shrunked fibrotic area and strengthened cardiac function	[172]
hydrogel	hUC-MSCs	MI model rat	Gel@Exo(a hyperbranched epoxy macromer (EHBPE) with hUC-MSC-EVs)	enhanced LVEF and FS, reduced fibrosis area	[158]
hydrogel	dendritic cells	MI model mouse	dendritic cell-derived exosomes (DEXs) in alginate hydrogel (DEXs-Gel)	improved cardiac function	[173]
hydrogel	BM-MSCs	MI model rat	MSC-sEVs in alginate hydrogel (sEVs-Gel)	improved cardiac function, reduced MI size	[90]
hydrogel	endothelial progenitor cells	MI model rat	shear-thinning hydrogel with EVs	improved left ventricular contractility, preserved global ventricular geometry	[93]
hydrogel	hUC-MSCs	MI model rat	hUC-MSC-EVs in functional peptide hydrogel	improved the myocardial function by reducing inflammation, fibrosis and apoptosis by promoting angiogenesis	[132]
hydrogel	iPSCs	MI model rat	implanted engineered hydrogel patch with EVs	reduced arrhythmic burden, promoted ejection fraction recovery, decreased cardiomyocyte apoptosis 24 h after infarction, reduced infarct size and cell hypertrophy 4 weeks post-infarction	[91]
hydrogel	endothelial progenitor cells	MI model rat	shear-thinning hydrogel with EVs	enhanced peri-infarct angiogenesis and myocardial haemodynamics	[92]
scaffold	cardiac adipose tissue-derived MSCs (cATMSCs)	MI model pig	engineered cardiac scaffolds with cATMSC-EVs in hydrogel	improved RVEF, inhibited left ventricular dilatation, reducted scar size	[174]
scaffold	cardiac adipose tissue-derived MSCs (cATMSCs)	MI model pig	engineered cardiac scaffolds with cATMSC-EVs	increased in vascular density, reduced in macrophage and T cell infiltration within the damaged myocardium	[175]
spray	MSCs	MI model mouce/pig	minimally invasive exosome spray (EXOS)	improved cardiac function, reduced fibrosis, promoted endogenous angiomyogenesis	[160]

EV: extracellular vesicle, DDS: drug delivery system, CPCs: cardiomyocyte progenitor cells, CDCs: cardiosphere-derived cells, BM-MSCs: bone marrow-mesenchymal stem cells, iPSCs: induced pluripotent stem cells, hUC-MSCs: human umbilical cord-mesenchymal stem cells, MI: myocardial infarction, IR: ischemia/reperfusion, TAC mouse: transverse aortic constriction mouse, MA-HA: methacrylated-hyaluronic acid, RGD: arginyl-glycyl-aspartic acid, LVEF: left ventricular ejection fraction, FS: fractional shortening, RVEF: right ventricular ejection fraction

### Administration of EVs Mixed Hydrogels or Other Drugs

The next challenge is to effectively deliver and retain EVs, enhanced by the loading of therapeutic additives, to the site of cardiac damage. EVs are combined with hydrogels or sprays to increase EV retention in the local area of the heart. Additionally, directly administering them locally is expected to have the effect of prolonged local retention of EVs.

Regional infusion of miRNA-181a-overexpressing MSC-EVs for MI suppresses the inflammatory response, increases the Treg cell ratio through inhibition of c-Fos protein, and promotes recovery of the infarcted heart [153]. However, injected EVs may be rapidly eliminated by the body, as, in one study, they were no longer detectable 3 h after myocardial injection [154]. To prevent this rapid elimination, MSC-EVs were encapsulated in functional peptide hydrogel and injected into the myocardial infarct border region of rats. The results showed prolonged EV preservation at the myocardial injection site, reduced apoptosis, inflammation, and fibrosis after MI, and improved cardiac function compared to the group injected with EVs alone [155]. Hu *et al*. demonstrated that genetically engineered ISL1-MSC-EVs overexpressing islet-1 (ISL1) have endothelial-protective and angiogenesis-promoting effects. Furthermore, they found that the combined use of angiogenin-1 hydrogel significantly maintained ISL1-MSC-EVs in the ischemic region, improving endothelial cell survival and angiogenesis and enhancing MI recovery [156]. Cheng *et al*. constructed an injectable ExoGel by embedding MSC-EVs in a hyaluronic acid hydrogel; injection of ExoGel into the pericardial cavity of TAC mice reduced left ventricular chamber size and prevented thickening of the left ventricular wall thickness. The feasibility and safety of ExoGel injection were further confirmed in a porcine model [157].

Yang *et al*. constructed hUC-MSC-EVs binding an injectable conductive hydrogel (Gel@Exo). Gel@Exos effectively prolonged EV preservation in ischemic myocardium, increased l left ventricular ejection fraction, reduced fibrotic areas, and greatly improved cardiac function after being injected into the injured rat heart. Gel@Exos also promoted cell proliferation and angiogenesis and had a marked therapeutic effect on postmyocardial infarction I/R [158].

Cheng *et al*. combined cardiac patch formation with intrapericardial injection of biocompatible hydrogels containing EVs to achieve minimally invasive delivery of therapeutic agents into the pericardial cavity for cardiac repair. Cardiac patches based on intrapericardial injection showed strong cardiovascular repair performance and improved cardiac function in both mouse MI models and clinically relevant porcine models [157, 159]. Thus, MSC-EVs in functional peptide hydrogels enhanced the safety and maintenance of EVs, suggesting that MSC-EVs in functional peptide hydrogels may be a practical and effective method for utilizing EVs in myocardial regeneration therapy.

Yao *et al*. produced a minimally invasive exosome spray (EXOS) based on MSC-EVs and biomaterials. The treatment involves spraying a mixture of MSC-EVs, thrombin, and fibrinogen on the surface of the infarcted myocardium by injection into the epicardium. In a mouse model of acute MI, EXOS inhibited fibrosis, promoted endogenous vascular myogenesis in the injured heart, and improved cardiac function. Furthermore, EXOS has been shown to be a promising strategy to deliver therapeutic EVs for cardiac repair in a porcine model [160].

### Expression of Target Specific Peptides or Antibodies on the Surface of EVs

Treatment with a hydrogel or spray in combination with EVs certainly increases the retention of EVs in the heart, but both require regional injection and are invasive, making them difficult to apply clinically. Intravenous injection of EVs, on the other hand, is a relatively minimally invasive method of treatment that can be easily applied clinically. Although EVs are said to have a certain degree of target specificity, their short *in vivo* half-life and rapid clearance make the intravenous injection of EVs insufficient for the treatment of target lesions. Therefore, many homing peptides have been discovered that target diseased tissues and organs, and homing peptides that are decorated with EVs and promote targeting to damaged tissue areas have been proposed.

Ciullo *et al*. constructed CPCs overexpressing CXCR4, which binds to stromal cell-derived Factor 1 (SDF-1α). SDF-1α is overexpressed in ischemic tissue, including infarcted myocardium. Intravenous injection of CXCR4-overexpressing CPC-EVs into rat models of MI resulted in a reduction in infarct size and improved left ventricular ejection fraction compared to injection of unmodified EVs. This effect was attributed to the accumulation of CXCR4-overexpressing CPC-EVs in SDF-1-rich infarcted myocardium, and their contents were effectively taken up by the infarcted myocytes [161]. Zhang *et al*. reported that MSC-EVs were modified with monocyte mimics through membrane fusion to improve the delivery efficiency of EVs to ischemia-injured myocardium. The monocyte membranes (Mons) were isolated from RAW 264.7 cells with commercial kits. MCS-EVs with Mons were cocultured and then passed through a polycarbonate membrane to obtain monocyte mimic-bioinspired MSC-EVs (Mon-Exos). Mon-Exos exhibited enhanced targeting efficiency to injured myocardium by mimicking the recruitment feature of monocytes after I/R. This strategy allowed Mon-Exos to promote endothelial maturation during angiogenesis and regulate macrophages after I/R, ultimately improving cardiac function in I/R model mice [162]. Focusing on the high expression of cTnI in the myocardial infarction region microenvironment, Wang *et al*. designed EV exosomes containing ischemic myocardial target peptides. To increase the accumulation of MSC-EVs carrying hsa-miRNA-590-3p, which promotes cardiomyocyte proliferation, at the MI region, cTnI-targeted EVs were created by expressing a cTnI-targeted short peptide on the surface of MSCs by transfection. These EVs were able to localize to the infarction region along the cTnI concentration gradient, and hsa-miRNA-590-3p-loaded MSC-EVs were endocytosed by cardiomyocytes, promoting peri-infarction myocyte proliferation and ultimately restoring cardiac function [163]. In addition, molecular cloning and lentivirus packaging technology was used to engineer exosomal enriched membrane protein (Lamp2b) fused with ischemic myocardium-targeting peptide CSTSMLKAC (IMTP). Compared with blank exosomes, IMTP-exosomes were observed to be increasingly accumulated in the ischemic heart region; as a result, attenuated inflammation and apoptosis, reduced fibrosis, enhanced angiogenesis, and cardiac function were detected by MSC-IMTP-EVs treatment in the ischemic heart region [164]. Other peptide motifs that bind preferentially to ischemic cardiac tissue include CKPGTSSYC and CPDRSVNNC [165, 166].

Liu *et al*. developed a novel strategy using magnetic nanoparticles to accumulate EVs in the region of MI treatment. Two types of antibodies (GMNPECs) were designed on the surface of these magnetic nanoparticles, which can bind to both the CD63 antigen, an EV surface marker, and myosin light chain surface markers of injured cardiomyocytes. By applying a regional magnetic field, therapeutic EVs captured by GMNPECs actively accumulate in the infarcted heart region. In rabbit and rat models of MI, EVs accumulated in damaged cardiac tissue and induced a reduction in infarct size, enhanced angiogenesis, and improved cardiac function [167].

## Conclusion

This article focuses on the latest findings on EVs as DDSs in the cardiac field. Although EVs tend to be focused on oncology, there are many reports of cardioprotective EVs as described above. Only a few effective drugs have been developed in recent years for the treatment of cardiovascular diseases, so one cannot help but have high expectations for EVs. Similarly, cell-based regenerative medicine has shown great promise for cardiac disease, but compared with cell-based therapy, EVs offer several potential advantages. The potential advantages of EVs over cell-based therapy include EV stability, relative non-immunogenicity, and capacity for pre- and post-isolation modification [176]. Furthermore, we have the potential to benefit from cell-free therapy with EVs without the ethical restrictions that would be regulated with ES cells. In addition, we also believe that the heterogeneity of EVs, which has been the focus of much attention in recent years, may be an advantage in treating cardiac diseases. No matter how precisely we recover EVs today, there are no completely the same EVs such as clones. The size and surface proteins of EVs and the composition of their inclusions differ slightly, and the inclusions usually contain nucleic acids, cytokines, and proteins with various bioactivities rather than a single chemical compound. We feel that such heterogeneity of EVs may have the potential to be effective in the treatment of lifestyle-related diseases such as cardiac diseases, which are caused by the accumulation of several factors. On the other hand, the most critical challenge of EV-based therapy in the field of cardiac diseases is the specificity of EVs to the heart and their retention. Most of the currently reported therapeutic effects of EVs on cardiac diseases are based on the direct administration of EVs into the myocardium or pericardial cavity, which is too invasive for clinical application [90–93, 132, 156–160, 172–175]. In order to achieve the therapeutic effect of EVs for cardiac disease by intravenous administration, which is a less invasive method of administration, it is essential to further improve such techniques as described above [section: Expression of Target Specific Peptides or Antibodies on the Surface of EVs]. However, the clinical application of EVs as DDS will not be realized without technological breakthroughs in the basic isolation method of EVs and in the encapsulation technology of many pharmacological compounds. There are still many things about EVs that remain to be revealed, but we believe that they are a very high potential therapeutic tool. We hope that this review will help in understanding EV research in the field of cardiac disease, and we look forward to the development of further EV research and effective therapeutic applications in cardiac disease.

## Data Availability

Data sharing not applicable to this article as no datasets were generated or analysed during the current study.
